# Content Comparison of Aquatic Therapy Outcome Measures for Children with Neuromuscular and Neurodevelopmental Disorders Using the International Classification of Functioning, Disability, and Health

**DOI:** 10.3390/ijerph16214263

**Published:** 2019-11-02

**Authors:** Javier Güeita-Rodríguez, Lidiane Lima Florencio, José Luis Arias-Buría, Johan Lambeck, Cesar Fernández-de-las-Peñas, Domingo Palacios-Ceña

**Affiliations:** 1 Department of Physical Therapy, Occupational Therapy, Rehabilitation and Physical Medicine, Rey Juan Carlos University, Alcorcón, 28922 Madrid, Spain; lidiane.florencio@urjc.es (L.L.F.); joseluis.arias@urjc.es (J.L.A.-B.); cesar.fernandez@urjc.es (C.F.-d.-l.-P.); domingo.palacios@urjc.es (D.P.-C.); 2 Research Group of Humanities and Qualitative Research in Health Science of Universidad Rey Juan Carlos (Hum&QRinHS), Rey Juan Carlos University, 28922 Madrid, Spain; 3 Association International Aquatic Therapy Faculty, CH-7317 Kliniken Valens, Switzerland; lambeck@freeler.nl

**Keywords:** outcome measure, content comparison, physical therapy modalities, International Classification of Functioning, Disability and Health, nervous system diseases

## Abstract

Currently, aquatic exercise is among the most common physical activity modalities for children with neuromuscular and neurodevelopmental disorders. However, the outcome measures that should be routinely used by therapists working in this specific health-care context have not been widely studied. The purpose of the study was to identify and compare the content of outcome measures used in aquatic physiotherapy for children, employing the International Classification of Functioning, Disability and Health (ICF) as a framework. A literature review was used to identify aquatic functioning outcome measures for children with neuromuscular and neurodevelopmental disorders. Content comparison of outcome measures identified was linked to the ICF following the linking guidelines, and content-related metrics were used to analyze them. Four outcome measures were identified (HAAR, Conatser, WOTA 1 and 2, and SWIM), which contained a total of 116 meaningful concepts and were linked to 35 ICF 2nd level categories. The greatest number of items assessed activity and participation categories. Large differences were found in reference to the density of content. For content diversity, the measures were all below 0.5. The identified outcome measurements showed homogeneity with respect to the theoretical foundation; however, some differences were found in terms of content analysis.

## 1. Introduction

Aquatic physiotherapy (APT) is defined as ‘the special practice of physiotherapy, with the therapeutic intent toward rehabilitation or attainment of specific physical and functional goals of individuals using the medium of water’ [1]. APT has a wide range of therapeutic and clinical effects, with improvements in gross motor performance, walking parameters, and gait efficiency in children with cerebral palsy (CP) [2,3]. Similar benefits are seen in motor skills of children with developmental coordination disorders (DCD) [4] and Duchenne muscular dystrophy (DMD) [5]. APT provides a motivating environment that promotes physical participation, increasing the functioning of children with neuromuscular disorders and other developmental disabilities. Studies suggest APT treatments can generate a positive effect on the behavior of children with autism spectrum disorders (ASD), encouraging participation and enhancing the adherence to treatments [6,7]. Aquatic therapeutic programs are often more propitious to participation and independent activities than many conventional land-based therapies.

When measuring the effectiveness of an intervention program, one needs to choose appropriate outcome measures. A lack of clarity in terms of functional areas to be included and measurement constructs can make the selection of outcome measures based on a recognized theoretical foundation challenging. There needs to be a clear description of the construct, and the theory on which it is based [8]. An exhaustive evaluation and examination of outcomes is necessary to fulfil this requirement [9,10]. While the psychometric properties and the application features of the measurements tools need to be taken into account, it is also important to look at the conceptualization of constructs and the content used to measure them [11]. Content analysis of the outcome measures based on a systematic and standardized method for linking the content of items with domains of a conceptual model (linking process) [12], like the International Classification of Functioning, Disability and Health (ICF) [13], would be valuable for content and face validity. This process may serve as a guide to select the most appropriate functioning measurement tools in APT treatment, in order to facilitate their use and implementation in practice, and for education and research by clinicians.

‘Functioning’ is defined by the ICF as ‘an umbrella term to describe what a person with a health condition does or is able to do in everyday life’ [13]. The purpose of the ICF is to create a framework for evaluating the pertinent domains of functioning and health. This is a useful classification that can be used to promote participation by children [14] and also identify relevant goals within professional interventions, such as physiotherapy [15]. In 2018, the ICF and the Children and Youth (CY) version of the ICF were merged into one [16], in order to address all aspects of functioning across the life-span of an individual [17]. The ICF addresses both what a person can do in a standard surrounding (their level of capacity) and what they do in their usual environment (their level of performance) [13].

The present study followed the ICF linking rules framework, established by Cieza et al. [18,19,20]. These rules have been used to link health-status and clinical assessments in different health-care contexts [21,22]. Thus, the ICF linking rules framework helps researchers and clinicians systematically compare health status information, clinical measures, and interventions inside ICF [15].

Aquatic exercise is currently amongst the most common physical activity modalities selected by children with CP and their parents [23], with APT often the first choice in addition to regular therapy [24]. However, the outcome measures of functioning that should be routinely used by physiotherapists working in the specific health-care context of APT have not been widely studied. In previous work by our group [25,26], we identified the relevant intervention categories available when using CY (‘what to measure’) from the perspective of parents and experts, to create an APT core set. The following step would be to identify which outcome measures (‘how to measure’) align with these categories, and also with the main neuromuscular and neurodevelopmental disorders core sets, such as for CP, ASD, and attention deficit hyperactivity disorder (ADHD) [27]. ICF could be connected with APT to solve this lack of an all-embracing structure to measure the functioning for those who participate in APT.

This study (1) identified the outcome measures more frequently used in APT with children and youth with neuromuscular and neurodevelopmental disorders, and (2) examined and compared the contents of these measurement tools, using the ICF as a framework.

## 2. Materials and Methods

A literature review with a methodological approach was conducted for original intervention studies that included children with neuromuscular and neurodevelopmental disorders receiving APT. The outcome measures that evaluated the aquatic functional skills were identified and the content comparison described using the ICF.

### 2.1. Identification of Outcome Measures

The following databases were searched for publications dating from 2008–2018: Cochrane, PEDro, CINAHL (Ovid), WOK, PubMed, EBSCO, Scopus, and SportDiscus. The MeSH terms and keywords used were ‘children’, ‘outcome measures’ OR ‘health status measurements’ and ‘aquatic therapy’. The outcome measurements were included if the publications reported specific aquatic therapy instruments, designed for children, that were available in English and had been tested for psychometric properties. Two researchers (J.G.-R. and D.P.-C.) conducted the inclusion process independently. The resulting sample of outcome measurements included the Conatser Adapted Aquatics Screening Test (Conatser) [28], Humphries´ Assessment of Aquatic Readiness (HAAR) [29], Swimming With Independent Measurement (SWIM) [30] and the Water Orientation Test Alyn (WOTA 1 and WOTA 2) [31].

### 2.2. Identification and Linking Process of Meaningful Concepts

We used the ICF as a tool to examine the aquatic functioning outcome measures in children with neuromuscular and neurodevelopmental disorders. To understand the linking process, it was important to have an insight into the ICF. Five components of functioning are included in the ICF classification: (1) body functions, (2) body structures, (3) activities and participation, (4) environmental factors, and (5) personal factors. These components consist of several chapters, with hierarchical ICF categories as the classification units. Altogether, the ICF components conformed to 1685 ICF-CY categories, excluding personal factors (unclassified to date). An alphanumeric code was assigned to each ICF category. This code represented the classification component with a letter (b, body functions; s, body structures; d, activities and participation; and e, environmental factors) followed by a number, which represented the chapter (e.g., b4). This was followed by a second level specification (e.g., b440). When applicable, a third- and fourth-level specification was also included (e.g., b4401).

### 2.3. The Linkage Process

Two health professionals (J.G.-R. and D.P.-C.), experienced with the ICF, performed the linking process separately. The items of the selected aquatic measures were examined to identify the meaningful concepts, defined as a ‘unit of text’, identified to convey a single theme based on the judgment of the health professionals and their expertise of functioning and the ICF [32]. For example, ‘perform a vertical/forward rotation’, an item from HAAR [29], contained the meaningful concept of ‘body position’ and was linked to d4104 standing. The concepts were linked to the most precise ICF category, following the linking rules proposed by Cieza et al. [18,19,20], which had been applied to a variety of pediatric outcome measures [11,33,34]. We documented the introduction, instructions, item response, and sociodemographic information (including personal data); however, these factors were not considered for the linking process. Only the aquatic functional skills items of the measurement tools were linked. Repeated concepts were taken into account only once.

Consensus between the two assessors was required for final linkage. A third assessor (J.L.) was available in cases of disagreement between the two main reviewers. If an item of the aquatic measures contained more than one meaningful concept, each concept was linked separately. Following the linking rules [18,19,20], items that were too general to warrant a code to a specific ICF category were considered as ‘nd-not defined’ (e.g., ‘adequate behavior’). If the concept described an aspect that was not covered by the ICF, it was labelled ‘nc-not covered’ (e.g., ‘buoyancy-floatation’). The domain ‘personal factors’ was not classified in the current version of the ICF, with meaningful concepts for this domain being coded as ‘pf’. No information was collected on the relationship between items and concepts (e.g., no differentiation between ‘backwards transversal rotation’ and ‘change position from standing to sitting position’), or subjective assessment within items (e.g., no differentiation between ‘maintains supine float’ and ‘static back float’). Finally, linked ICF categories were revised and compared to the brief ICF core sets for CP, ASD, and ADHD [27].

### 2.4. Analysis and Reliability

The analysis developed was content-based at the level of wording and phrasing. We calculated the frequency distribution of the meaningful linked concepts across the ICF components (b, s, d, and e) and pf, and other concepts linked to nc and nd, with each identified instruments. This data analysis allowed quantification of the most relevant areas of APT functioning for children. We used descriptive statistics to calculate the frequency with which a category was linked in each outcome measure. No cut-offs were used to exclude categories. The reliability of the linkage process was calculated with kappa coefficients and nonparametric bootstrapped confidence intervals [35].

### 2.5. Content Analysis

Content-related metrics were based on the data analysis of the linking process (bandwidth of content coverage, content density and content diversity). These metrics were developed and used to compare measures in adults with neurological disorders by Geyh et al. [36].

The bandwidth of content coverage was analyzed by the frequency distribution of categories across the four linked components of the ICF [36], and the percentage of the total ICF categories covered by the aquatic skills measures. SWIM, for example, was linked to 13 different ICF categories, covering 0.77% of all existing ICF categories.

Content density was a measure of multidimensionality within the item structure of an instrument. It was represented by a ratio of the number of meaningful concepts identified by the linking procedure divided by the total number of items in the measure [36]. Ratios close to 1 indicated that each item contained one meaningful ICF concept, while higher values showed that there were several concepts contained within each item. HAAR was found to have 40 concepts rejected in the 32 items, giving a content density ratio of 1.25.

Content diversity was a measure of reach of an instrument with regard to the ICF categories covered. It was the ratio of the number of 2nd level ICF categories divided by the number of meaningful linked concepts [36]. Values close to 1 indicated that each meaningful concept of the measure corresponded to a different ICF category. Values closer to zero meant that several meaningful concepts in the measure related to the same ICF category. For example, in the WOTA 2, 16 different 2nd level ICF categories were needed in order to draft the 60 concepts, giving a content diversity ratio of 0.26.

These metrics offered an additional estimate of the content of the scales. They helped to partially answer the question of how it was measured in a discipline. They could also be useful when choosing the most effective outcome measures, although they should not be the only aspect to consider.

## 3. Results

### 3.1. Overview of Outcome Measures

Four aquatic functioning outcome measures were identified and included in the content comparison. One of them was double (WOTA 1 and 2) [31], as it had two versions depending on the situation and age of the child. For these five measures, their main characteristics in terms of format, theoretical foundations, target populations, as well as their psychometric properties are shown in Table 1.

### 3.2. Linking Process

Overall, the four outcome measures contained 127 items. A total of 116 meaningful concepts were identified and linked during item analysis. SWIM [30] contained the highest (*n* = 28) and the WOTA 1 [31] contained the lowest (*n* = 18) number of meaningful concepts. The results of the linking process are shown in Table 2, enumerating meaningful concepts, linked concepts to ICF categories and the outcome measure metrics. The 116 meaningful concepts were linked to 35 ICF categories based only on domains described in ICF. The estimated kappa value was 0.65 (bootstrapped confidence interval, 0.54–0.79)

### 3.3. Examination of ICF-Based Content

None of the outcome measures addressed all the ICF domains. All measures contained body functions categories (seven categories), except the HAAR (Table 2) [29]. By contrast, the body structures component was not covered by any measure. For the activities and participation component (24 categories), all the outcome measures covered categories, being the most frequent component. Environmental factors (four categories) were just included in the Conatser and SWIM. Personal factors and other factors (120 meaningful nc concepts and 36 nc categories) following linking rules [18,19,20] (rule 3, nd + nc, and health conditions) are included in Table 2 for descriptive use, but they are not linked in Table 3.

Table 3 shows the number of categories from extracted concepts per ICF component, with the second level out of the total being presented. None of the linked categories were included in the four scales at the same time. There were only three categories present in all the outcome measures: d410, changing basic body positions; d4401, grasp; and d4554, swimming. The ‘breathing’ concept was linked to categories b440, b4400, b4401, and b4408, depending on the general or specific content of the item, since some measures referred to breathing in general, while in other cases it was a qualifying property, such as in the WOTA 2 with frequency and rhythm [31]. For the mobility chapter, the largest number of linked responses referred to ‘functions related to mobility’ (b710), ‘functions related to force’ (b730), and ‘functions related to the reflexes of involuntary movements’ (b755), and each was linked in only two measurements.

For the activities and participation component, all the linked categories were included in chapter 4 of mobility, except for ‘free time and leisure’ (d920), which was covered by the HAAR [29]. Notably, within the mobility chapter, ‘change basic body positions’ (d410), ‘grasping’ (d4401), and ‘swim’ (d4554) showed the maximum response, each being named by at least five of the aquatic functioning measures (80%).

The outcome measures Conatser [28] and the SWIM [30], dividing the number of items equally, were the only ones that covered the environmental factors component.

The WOTA 2 was the outcome measure with the broadest bandwidth of content coverage, with 16 ICF categories. The outcome measures with the narrowest bandwidth of content coverage were the HAAR and WOTA 1 (11 categories). The SWIM showed the highest value for content density (5.09), with 11 items containing 56 meaningful concepts. Conatser showed the lowest content density (1.09), with 44 items containing 48 meaningful concepts. The SWIM showed the lowest ratio for content diversity (0.23), using 13 ICF 2nd level categories to map 56 meaningful concepts. The content diversity was highest in the Conatser (0.31), with 15 different ICF categories representing 48 concepts.

## 4. Discussion

This study provides a comprehensive overview and comparison of currently used aquatic functioning measures in children with neuromuscular and neurodevelopmental disorders. It also characterizes the content related to aquatic skills in each of the analyzed measures based on its ICF representation. Our findings show that the greatest number of items focused on assessing activity and participation. Body functions, environmental factors, and personal factors were less frequently represented, as were other factors not recognized by the ICF. These results suggested a poor representation of the personal features of children and the implicit aquatic environment (mechanical constraints) with respect to important interactions with the increased functional status of the child in the water. This could indicate there is a need for an assessment that determined functional gain in daily life based on APT, because there are many outcomes within aquatic environment that are transferred to the daily lives of children.

The identified outcome measures showed homogeneity with respect to the theoretical foundation, overlapping some ICF components. Overall, they provided a clear definition of their theoretical foundation, because their approach to aquatic functioning was based on the same Halliwick concept [37].

The content metrics of the identified outcome measures partly answered the question of how measurements were currently made in the field of aquatic therapy for children with neuromuscular and neurodevelopmental disorders, which was helpful for the selection of those that were the most efficient. Small differences were found between the outcome measures included in our study in reference to the density of content, with them covering almost the same aspects of activities, even when measures differed by length and number of concepts included. HAAR and Conatser were the outcome measures with the lowest levels of content density, showing less complex items, which might be more applicable in clinical settings. On the other hand, the SWIM had the highest content density, with items that measure more than one concept. Gradinger suggested that the denser outcome measures should be combined with less dense measures in order to provide a broader spread of assessment [38].

With respect to content diversity, the outcome measures had low levels (all below 0.5). This means they were very tight measures of the concept they were evaluating, including several concepts for the same topic (in this case, aquatic skills). HAAR and WOTA 1 showed the smallest bandwidth of content coverage. This might suggest that both were focused on few but relevant domains. On the other hand, Conatser and WOTA 2 had the greatest bandwidth, comprising items from a higher number of different domains.

The figures of content density, diversity, and bandwidth of content coverage of a measure do not necessarily mean they were better or worse measures. These metrics make the outcome measures comparable with regard to ICF-based content covered. Furthermore, these indices might guide the selection of measures with potential for refinement in clarity or redundancy. They might also help in identifying those outcome measures suitable for translation into different languages and use in international studies, based on their content structure [36].

The selection of a suitable outcome measure is based on many factors, such as the content properties described in results, but also other psychometric properties. Considering the psychometric properties of the aquatic measures, WOTA 1 and 2 had the highest number of properties tested compared to the other outcome measures, most of which only assessed the validity of content and inter-observer reliability. Reliability of measures was acceptable, with intraclass correlation coefficients (ICC/kappa) >0.70.

Another important factor was the correlation with outcome measures on land, where WOTA 1 and 2 were the only ones that showed such a correlation. As there is no available established gold standard for evaluating adjustment and function in the aquatic environment, the concurrent validity was verified using outcome measures that assess these aspects on land. A moderate concurrent validity was confirmed by significant and positive correlations between WOTA 1 total score and the Brief Assessment of Motor Function Test (r = 0.56), and between WOTA 2 and the Gross Motor Function Measure (r = 0.60) [39].

In assessing which measurement to use, it is also important to determine their applicability, and the time needed for their application was fundamental. The average application time of all measures was 15–30 min. A final consideration for the selection of outcome measures was the prior training needed to use them. In this case, the WOTA 1 and 2 were the only ones that defined this concept as a prerequisite, ensuring its correct application.

The measurements included in this current study measured mainly aquatic skills, being based on the same Halliwick concept [37], and subdivided according to whether the outcome measure focused more on therapy or on aspects related to the learning of swimming. The overview provided by our study indicated the simplicity of the aquatic assessment as a unidimensional field of evaluation.

The results of the current study could be compared to existing ICF core sets in children with neuromuscular and neurodevelopmental disorders, such as CP, ASD, and ADHD [27]. The activities and participation categories were the most covered across the three core sets in the current study, reflecting the impact of the three conditions on broad areas of day-to-day functioning. The aquatic functioning measures content comparisons were aligned with the three core sets as b7, neuromusculoskeletal and movement-related functions, and d4, mobility chapters. There was also consensus regarding e1, products and technology, e4, attitudes, and e5, services, systems and policies, showing that water was a facilitator factor for assistive technologies and attitudes in many aspects of functioning in children and young people with CP. However, most of these functioning chapters of the three core sets were not included in our findings, being suggested as areas that might be included in future aquatic functioning assessments for children with neuromuscular and neurodevelopmental disorders. Regarding body functions, the main chapter not covered in our study was the b1 mental functions chapter, which was more often covered in the ADHD and ASD ICF core sets compared to CP. For activities and participation, d1 learning and applying knowledge was the chapter most frequently covered in the three core sets. Finally, for environment factors, e3 support and relationships was not influenced by APT. An important chapter which did not match with ASD and ADHD was b4, functions of the cardiovascular, hematological, immunological, and respiratory systems; however, it was covered in the CP core set.

The limitations of this study were fundamentally in conceptualization, since the linking rules used in this content comparison did not differentiate between the concepts and contexts included in an item. In this study, it was especially important, due to the aquatic context, where the outcome measures were applied. In many cases, categories not included in the ICF had to be used for specific concepts of the aquatic environment included in the outcome measures. Therefore, several meaningful concepts were not covered within ICF, which might have resulted in loss of information from the outcome measures.

## 5. Conclusions

The use of the ICF as an external template to compare the contents of aquatic skill measures revealed homogeneity with respect to the theoretical foundation; however, it also showed some differences in the content analysis. The comparison of aquatic functioning measures provided novel information with respect to the precision of their specific concepts related to aquatic skills, which was useful when planning and selecting outcomes measures for future studies. This knowledge of the content of outcome measures could help to capture specific information regarding skills that researchers and clinicians want to examine in water. However, it would also be advisable to add categories included in the main functioning neuromuscular and neurodevelopmental ICF core sets to future aquatic functioning assessments, in order to develop outcome measures that determine functional gains in daily life based on APT.

## Figures and Tables

**Table 1:** Overview of the aquatic functioning outcome measures.

Measure	Abbreviation	Format	Aquatic Skills Phases	Population in Psychometric Studies	Psychometric Properties Studied
Adapted Aquatics Screening Test	Conatser [28]	44-items in 6 skills	Adjustment Skills, entering/exiting the pool, Range of movement, Breath control, Balance and flotation and Active movement in water. Entering and Exiting the Pool	CP, ASD and motor retardation, Age 5–21	Intrarater reliability (ICC = 0.83)
Humphries’ Assessment of Aquatic Readiness	HAAR [29]	32-items in 5 phases. Rating “0” (unable) and “1” (able)	Mental Adjustment, Introduction to water environment, Rotations, Balance and Controlled Movement and Independent movement in water	ASD Age 3–17	Interrater reliability (ICC = 0.93)
Swimming With Independent Measurement	SWIM [30]	11 items. Rating done on a seven-point scale (7 is the best result).	Water entry, Water adjustment, Breath control, Balance, Backward transversal rotation, Forwards transversal rotation, Sagittal rotation, Longitudinal rotation, Combined rotation. Water stroke and Exit	ASD, CP, Chromosomopathy, Down syndrome, Myelomeningocele, Mental retardation Age 7–22	Interrater reliability (ICC = 0.99)
					Face validity (SWIM and NESSA are related; *p* < 0.001)
The Water Orientation Test Alyn 1	WOTA 1 [31]	13-items Rating done on a 1–4 scale (4 is the best result)	General Adjustment, Entry/exit from pool, Blowing bubbles, Side/Back floating, “Splashing”, Submerging, Vertical position, Progress along Wall, Standing, Holding rope and Sitting.	CP and other neuromuscular disorders Age 3–13	Test-retest reliability (ICC = 0.97; MDC = 4.2)
					Concurrent validity (BAMF and WOTA 1 score, rP = 0.56, *p* < 0.05)
The Water Orientation Test Alyn 2	WOTA 2 [31]	27-items in 4 sections Rating done on a 0–3 scale (3 is the best result)	Mental Adaptation, Balance control and Movement	CP and other neuromuscular disorders Age 3–13	Test-retest reliability (ICC = 0.97, MDC = 11.5)
					Concurrent validity (GMFM and WOTA 2 function subtest score, rP = 0.6, *p* < 0.05)

CP: Cerebral Palsy; ASD: Autism Spectrum Disorder; NESSA: National Evaluation System of Swimming Abilities; GMFM: Gross Motor Function Measurement; BAMF: Brief Assessment of Motor Function Test; rP: Pearson’s correlation coefficients; ICC = intraclass correlation; MDC = minimal detectable change.

**Table 2:** Linking process and content-related metrics.

Content-Related Metrics	Total	CONATSER	HAAR	SWIM	WOTA 1	WOTA 2
Number of items of each measure	127	44	32	11	13	27
Number of meaningful concepts covered	116	26	20	28	18	24
Number of meaningful concepts non covered	120	22	20	28	20	26
Total of meaningful concepts	236	48	40	56	38	60
Content density *	1.85	1.09	1.25	5.09	2.92	2.22
Categories linked to the 2nd level ICF	35	15	11	13	11	16
Bandwidth (%, *N* = 1685)	2%	0.89%	0.65%	0.77%	0.65%	0.94%
Content diversity **	0.14	0.31	0.27	0.23	0.28	0.26
Body Functions categories (*n*)	7	3	0	2	3	4
Activity and Participation categories (*n*)	24	10	11	9	8	12
Environmental Factors categories (*n*)	4	2	0	2	0	0
Categories non covered by ICF	36	11	10	14	10	22

* Calculated as Content Density (=meaningful concepts/n outcome measure items) and ** Content Diversity (=*n* of 2nd level ICF categories/meaningful concepts).

**Table 3:** The International Classification of Functioning, Disability, and Health-ICF categories.

ICF Code	ICF Categories	CONATSER	HAAR	SWIM	WOT1	WOTA2	%
Body Functions							
b440	Respiration functions			x	x	x	60.00
b4400	Respiration rate					x	20.00
b4401	Respiratory rhythm					x	20.00
b4408	Respiration functions, other specified	x					20.00
b710	Mobility of joint functions	x		x			40.00
b730	Muscle power functions	x			x		40.00
b755	Involuntary movement reaction functions				x	x	40.00
Activities and participation							
d410	Changing basic body position	x	x	x		x	80.00
d4100	Lying down		x	x		x	60.00
d4103	Sitting					x	20.00
d4104	Standing		x	x			40.00
d4105	Bending			x			20.00
d415	Maintaining a body position		x	x		x	60.00
d4150	Maintaining a lying position		x				20.00
d4153	Maintaining a sitting position		x		x	x	60.00
d4154	Maintaining a standing position	x		x	x		60.00
d420	Transferring oneself				x	x	40.00
d435	Moving objects with lower extremities	x					20.00
d4351	Kicking	x	x				40.00
d440	Fine hand use	x		x			40.00
d4401	Grasping	x	x		x	x	80.00
d445	Hand and arm use	x			x	x	60.00
d4451	Pushing					x	20.00
d4452	Reaching		x				20.00
d446	Fine foot use	x					20.00
d455	Moving around	x		x			40.00
d4551	Climbing				x		20.00
d4554	Swimming	x	x	x		x	80.00
d460	Moving around in different locations				x	x	40.00
d465	Moving around using equipment				x	x	40.00
d920	Play		x				20.00
Environmental context							
e110	Products or substances for personal consumption			x			20.00
e115	Products and technology for personal use in daily living			x			20.00
e450	Individual attitudes of health professionals	x					20.00
e580	Health services, systems and policies	x					20.00
